# Vitamin E TPGS 1000 Induces Apoptosis in the K562 Cell Line: Implications for Chronic Myeloid Leukemia

**DOI:** 10.1155/2021/5580288

**Published:** 2021-06-09

**Authors:** Jazmin Calvo-Alvarez, Marlene Jimenez-Del-Rio, Carlos Velez-Pardo

**Affiliations:** Neuroscience Research Group, Medical Research Institute, Faculty of Medicine, University of Antioquia (UdeA), Calle 70 No. 52–21, and Calle 62 # 52–59, Building 1, Room 412; SIU Medellin, Colombia

## Abstract

Chronic myeloid leukemia (CML) is a hematologic malignancy derived from the myeloid lineage molecularly characterized by t(9;22)(q34;q11) resulting in BCR-ABL1 gene fusion, which is known as Philadelphia (Ph) chromosome. Although tyrosine kinase inhibitors (TKIs) have restored and maintained the quality of life of patients with CML, an important minority of patients become resistant to first-and-second-generation TKIs and require an alternative treatment. The K562 cell (Ph+, p53-/-) line was treated with Vit E TPGS 1000 (20–80 *μ*M) only or with other products of interest (e.g., antioxidant N-acetylcysteine (NAC), specific JNK and caspase-3 inhibitor SP600125, and NSCSI, respectively) for 24 h at 37°C. Cells were analyzed by fluorescence microscopy (FM), flow cytometry (FC), and Western blotting (WB) techniques. We show that TPGS induces apoptosis in K562 cells through H_2_O_2_ signaling mechanism comprising the activation of a minimal molecular cascade: the kinase JNK>the transcription factor c-JUN>the activation of BCL-only BH3 proapoptotic protein PUMA>loss of mitochondrial membrane potential (*ΔΨ*_m_)>activation of caspase-3>chromatin condensation>fragmentation of DNA. Additionally, TPGS oxidizes the stress sensor protein DJ-1-Cys106-SH into DJ-1-Cys106-SO_3_ and arrested the cell cycle in the S phase. Remarkably, NAC, SP600125, and NSCSI blocked TPGS-induced OS and apoptosis in K562. Since TPGS is safe in mice and humans, it is especially promising for preclinical and clinical CML leukemia research. Our findings support the view that oxidation therapy offers an important opportunity to eliminate CML.

## 1. Introduction

Chronic myeloid leukemia (CML) is a myeloproliferative neoplasm of the bone marrow [1] characterized by a genetic alteration known as chromosome Philadelphia (Ph), which consists of the fusion of the Abelson murine leukemia (*ABL1*) gene on chromosome 9 with the breakpoint cluster region (*BCR*) gene on chromosome 22 [2]. This gene fusion results in the expression of an oncoprotein termed BCR-ABL1 [3, 4]. According to the American Cancer Society (https://www.cancer.org/), there will be 9110 new cases diagnosed with CML, and 1220 people will die of CML in 2021. CML represents about 15% of all new cases of leukemia in the United States and mainly affects adults with an average age at diagnosis around 64 years, but rarely seen in children [5]. Even though the current first-, second-, and third-generation tyrosine kinase inhibitors (TKIs) are being used for treating patients with CML with great success [5, 6], still some patients (~10%-15%) fail to TKI treatment, due to mutations within the kinase domain of BCR-ABL1 protein [3, 7]. Therefore, it is urgent to investigate alternative therapeutic approaches such as natural product compounds and organic/synthetic new chemical entities [8–11] for CML treatment.

Vitamin E D-*α*-tocopheryl polyethylene glycol succinate (TPGS) is a synthetic derivative of natural *α*-tocopherol, prepared from the esterification of *α*-tocopheryl succinate (*α*-TOS) and polyethylene glycol (PEG) 1000 [12]. Although TPGS has FDA approvement as a safe adjuvant and is widely used in drug delivery systems as a single agent, it has proved effective as an anticancerogenic compound in human cells such as lung [13], prostate [14], breast [12, 15, 16], pancreatic [12], acute lymphoblastic leukemia (ALL) [17], neuroblastoma [18], and hepatocarcinoma [19] cancer cells. Although the mechanism of TGPS has not yet been fully elucidated, TPGS kills cancer cells through various mechanisms, including the inhibition of the activity of ATP-dependent P-glycoprotein overcoming multidrug resistance [12], by reactive oxygen species- (ROS-) induced apoptosis—a regulated cell death [17], cell cycle arrest, and apoptosis [16, 19], through both caspase-dependent and -independent DNA damage and dominant caspase-independent programmed cell death [16]. Studies on the effect of TPGS in leukemia cells are scarce [20]. Consequently, no data are available to establish whether TPGS induces apoptosis in CML and whether the mechanism of TPGS-induced cell death is similar to a ROS-induced signaling mechanism previously demonstrated in ALL [17]. Furthermore, it is not yet known whether BCR-ABL kinase confers CML resistance to TPGS stimuli. Therefore, to get insight into these issues, we have selected the K562 cell line as a model cell [21] to investigate the cytotoxic effect of TPGS. Indeed, the K562 is a well-characterized cell line [22] that expresses the Philadelphia chromosome, i.e., *BCR-ABL* [23, 24] responsible for maintaining proliferation, inhibiting differentiation, and conferring resistance to cell death [25]. Importantly, the K562 cell line does not express p53 [26, 27]—a transcription factor implicated in cell cycle regulation, chemical-induced OS response, and apoptosis [28, 29]. Therefore, it is imperative to answer whether K562 is resistant to TPGS exposure.

In this study, we determine for the first time that TPGS induces apoptosis in the K562 cell line mediated by the OS mechanism. The mechanism involves generating H_2_O_2_, the oxidation of the redox sensor DJ-1 protein into DJ-1-Cys106-SO_3_ (*sulfonate*) derivative; activation of the proapoptotic c-JUN transcription factor; the expression of PUMA; the loss of mitochondrial membrane potential (*ΔΨ*_m_); the activation of the protease caspase-3; and nuclear fragmentation, as apoptotic markers. We found that the BCL-2/BAX ratio was unaffected by TPGS in K562. It is also shown that the antioxidant *N-acetylcysteine* (NAC) and pharmacological inhibition of JNK (SP600123) and caspase-3 (NSCI) protect K562 against the cytotoxic effect of TPGS. In agreement with others, our findings support the use of TPGS as a treatment for patients with CML.

## 2. Material and Methods

### 2.1. Cell Line Conditions and Treatments

The K562 cells (ATCC Catalog No. CCL-243TM; Manassas, Virginia, USA) were cultured according to the provider's protocol. Briefly, a cryovial containing the frozen K562 cells was rapidly (<1 min) thawed in a 37°C water bath. Then, cells were incubated using a prewarmed growth medium composed of RPMI 16–40 medium with L-glutamine and sodium bicarbonate (cat. # R8758, Sigma-Aldrich, St Louis, Missouri, USA), fetal bovine serum (FBS) 10% and 100 U/mL penicillin, and 100 mg/mL streptomycin. When the cells were ready for passaging (i.e., log-phase growth before they reach confluency), they were subcultured. Cell suspensions at passages 3 to 5 were used for further experiments. The cell suspension (1 × 10^6^ cells/well in 1 mL final volume) was exposed to increasing *α*-tocopherol polyethylene glycol 1000 succinate (TPGS, CAS Number 9002-96-4, Sigma-Aldrich, St Louis, Missouri, USA) concentrations (10, 20, 40, 60, and 80 *μ*M). TPGS was prepared in PBS and stored to −20°C in the absence or presence of different products of interest (e.g., antioxidant and inhibitors) for 24 h at 37°C. The 3,30-dihexyloxacarbocyanine iodide (DiOC_6_(3)) (cat. # D-273, Thermo Fisher Scientific Inc.) and 1,9-pyrazoloanthrone (SP600125, cat. No. 420119) were purchased from Calbiochem (Merck Millipore). The dichlorofluorescein diacetate (DCFH_2_-DA) was from Invitrogen. Propidium iodide (PI) was acquired from BD Bioscience (San Jose, CA). All other reagents were from Sigma-Aldrich (St Louis, Missouri, USA).

### 2.2. Morphological Assessment of Cell Death by Fluorescence Microscopy

The cell suspension (1 × 10^6^ cells/well in 1 mL final volume) was exposed to increasing concentrations of TPGS for 24 h at 37°C. Fluorescence microscopy analysis was performed using a Zeiss Axiostart 50 Fluorescence Microscope equipped with a Zeiss AxioCam Cm1 (Zeiss Wohlk-Contact-Linsen, Gmb Schconkirchen, Germany). The adjustment of the images obtained was performed using the software provided by the manufacturer (ZEN 2 Core). The apoptotic indices were assessed two times in independent experiments blind to the experimenter.

### 2.3. Determination of DNA Fragmentation and Cell Cycle by Flow Cytometry

DNA fragmentation and cell cycle were determined using a hypotonic solution of PI according to ref. (30). After treatment, 1 × 10^5^ cells were washed with PBS (pH 7.2) and stored in 95% ethanol overnight at −20°C. Then, cells were washed and incubated in 400 *μ*L solution containing propidium iodide (PI, 50 *μ*g/mL), RNase A (100 *μ*g/mL), EDTA (50 mM), and Triton X-100 (0.2%) for 30 min at 37°C. The cell suspension was analyzed for PI fluorescence by using an Epics XL flow cytometer (Beckman Coulter). Quantitative data and figures were obtained using the FlowJo 7.6.2 Data Analysis Software. Cells entering the sub-g1 phase were used as a marker of apoptosis (DNA fragmentation). For cell cycle analysis, the sub-g1 population was subtracted from the total acquired events, and the Dean Jett Fox analysis was performed (RMS < 10). The experiment was conducted three times, and 10000 events were acquired for analysis.

### 2.4. Analysis of Mitochondrial Membrane Potential (*ΔΨ*_m_) by Flow Cytometry

To test the *ΔΨ*_m_, we incubated cells (1 × 10^5^ cells) for 20 min at RT in the dark with cationic and lipophilic 3,30-dihexyloxacarbocyanine iodide [DiOC_6_(3), 10 nM final concentration] compound (Calbiochem, Darmstadt, Germany; cat. No. D-273) according to ref. (30). The *ΔΨ*_m_ was measured by cellular retention of DiOC_6_(3), which is selectively taken up by mitochondria and reflects the maintenance of *ΔΨ*_m_ (ex. 450–490 nm, em. 515 nm). Cells were then analyzed using an Epics XL flow cytometer (Beckman Coulter). The experiment was conducted two times, and 10000 events were acquired for analysis using the FlowJo 7.6.2 Data Analysis Software.

### 2.5. Evaluation of Intracellular Hydrogen Peroxide Levels by Flow Cytometry

To determine intracellular H_2_O_2_ levels, we used 2′,7′-dichlorofluorescein diacetate (DCFH_2_-DA; Invitrogen) as described in ref. (17). Briefly, cells (1 × 10^5^) exposed to increasing *α*-tocopherol polyethylene glycol 1000 succinate concentrations (TPGS 10, 20, 40, 60, and 80 *μ*M) were then incubated with DCFH_2_-DA (5 *μ*M) reagent for 30 min at 37°C in the dark. Cells were washed, and DCF fluorescence was determined using an Epics XL flow cytometer (Beckman Coulter). The assessment was repeated two times in independent experiments. Quantitative data were obtained as described above.

### 2.6. Detection of Oxidized (Cys106-SO_3_) DJ-1 and Caspase-3 by Flow Cytometry

After each treatment with or without TPGS, cells (1 × 10^5^ cells/well) were fixed in 80% ethanol and stored at −20° C overnight. Then, cells were washed with PBS and permeabilized with 0.2% Triton X-100 plus 1.5% bovine serum albumin (BSA) in phosphate-buffered saline (PBS) for 30 min. Cells were washed and incubated with anti-PUMA (Abcam, cat. No. ab-9643) and caspase-3 (Rabbit, Millipore, cat. No. AB3623) primary antibodies (1 : 500, diluted in PBS containing 0.1% BSA). Subsequently, the cells were washed and incubated with (1 : 500) Dylight donkey anti-rabbit (594 nm, cat. No. DI-1094) or -mouse (488 nm, cat. No. DI-2488) secondary antibodies for 30 min at RT in the dark. After washing with PBS, the cells were suspended in 500 *μ*L of PBS. The analysis was performed on a BD LSRFortessa II flow cytometer (BD Biosciences). Cells without primary antibodies served as a negative control. For assessment, it was acquired 10000 events and quantitative data and figures were obtained using FlowJo 7.6.2 Data Analysis Software.

### 2.7. Western Blotting Analyses

K562 cells were left untreated or treated with different products of interest, and then, whole cells were lysed in 50 mM Tris-HCl, pH 8.0, with 150 mM sodium chloride, 1.0% Igepal CA-630 (NP-40), and 0.1% sodium dodecyl sulfate, 1 nM PMSF, and a protease inhibitor cocktail (Sigma-Aldrich). Forty micrograms of proteins in either reducing or nonreducing a loading buffer was loaded onto 12% electrophoresis gels and transferred to nitrocellulose membranes (Hybond-ECL, Amersham Biosciences) at 275 mA for 70 min by an electrophoretic transfer system (BIO-RAD). The membranes were incubated overnight at 4°C with primary antibodies. DJ-1 oxidation was determined by antibodies against cysteine sulfonic (SO_3_) acid (1 : 500; ox (Cys106)DJ-1; spanning residue C106 of human PARK7/DJ−1; cat. No. ab169520, Abcam), and proapoptotic molecules were determined by p-c-JUN (S63/73) (cat. No. sc-16312) and caspase-3 (Millipore, cat. No. AB3623) antibodies (1 : 5000). Mitochondrial death signaling was determined using BAX (cat. No. sc-493), BCL-2 (cat. No. 13–8800), and PUMA (cat. No. ab-9643) rabbit polyclonal antibodies. We used mouse anti-actin (cat. No. MAB1501, Millipore; 1 : 5000) antibody as an expression control. IRDye 800CW donkey anti-rabbit, IRDye 800CW donkey anti-goat, and IRDye 680CW donkey anti-mouse (LI-COR Biosciences; 1 : 10000) were used as the secondary probes. The blots were developed using the Odyssey Infrared Imaging System. The WB analysis was assessed two times in independent experiments.

### 2.8. Antioxidant and Pharmacological Experiments

K562 cell suspension (1 × 10^6^ cells/well in 1 mL final volume) was left untreated or treated with increasing *α*-tocopherol polyethylene glycol 1000 succinate concentrations (TPGS, 10, 20, 40, 60, and 80 *μ*M) only or along with either antioxidant N-acetyl-L-cysteine (NAC, 1 mM) or inhibitor reagents such as NSCI (1-(4-methoxybenzyl)-5-(2-(pyridin-3-yloxymethyl)pyrrolidine-1-sulfonyl)−1H-indole-2,3-dione, 10 *μ*M) and SP600125 (1,9-pyrazoloanthrone, 1 *μ*M) at 37°C for 24 h. The pharmacological inhibitor concentration was determined in previous experimental settings in our laboratory [17]. The cells were then evaluated for DNA fragmentation and *ΔΨ*_m_ by flow cytometry. The assessment was repeated two times in independent experiments.

### 2.9. Statistical Analysis

Statistical analyses were performed using the GraphPad Prism 6 scientific software (GraphPad, Software, Inc. La Jolla, CA, USA). Data are expressed as the mean ± SD of a minimum of two independent experiments. One-way ANOVA with a Tukey post hoc test was used to compare the differences between the experimental groups. A *p* value <0.05 (^∗^), <0.01 (^∗∗^), and <0.001 (^∗∗∗^) was considered statistically significant.

## 3. Results

### 3.1. TPGS Induces the Loss of Mitochondrial Membrane Potential (*ΔΨ*_m_), Cell Cycle Arrest, and DNA Fragmentation in Leukemia K562 Cells

**Figure 1:**
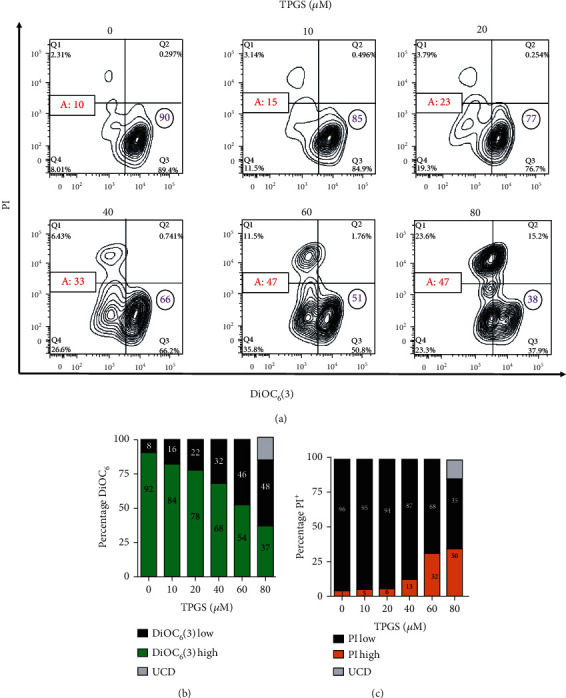
TPGS concentration-dependently induces nuclei fragmentation and loss of mitochondrial membrane potential (*ΔΨ*m) in K562 cells. K562 cells were left untreated (0 *μ*M) or treated with increasing concentrations of TPGS (10–80 *μ*M) at 37°C for 24 h. After this time, cells were double-stained with PI (ex. 535 nm, em. 615 nm) and DiOC_6_(3) (ex. 450–490 nm, em. 515 nm) stain. (a) Representative contour plot figures showing alive cells (Q3, PI^low^/DiOC_6_(3)^high^), early apoptotic (EA) cells (Q4, PI^low^/DiOC_6_(3)^low^), late apoptotic (LA) cells (Q1, PI^high^/DiOC_6_(3)^low^), and unspecific cell death, UCD (Q2, PI^high^/DiOC_6_(3)^high^). A: represents the percentage of apoptotic cells (Q1+Q4). The purple number in circles represents viable cells, and the red number in rectangles represents total apoptotic cells. (b) Representative histogram of DiOC_6_(3)^low/high^ and UCD assessed by flow cytometry. (c) Representative histogram of PI^low/high^ and UCD assessed by flow cytometry according to *Materials and Methods*.

**Figure 2:**
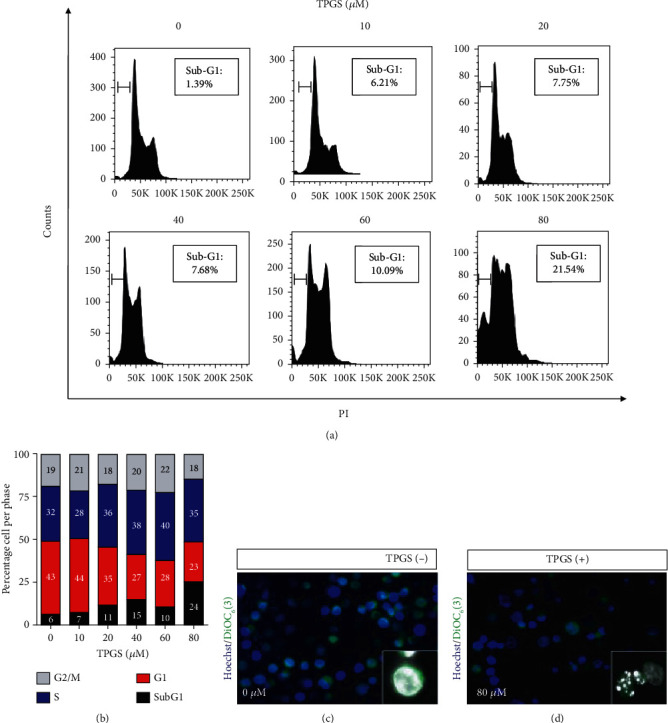
TPGS induces nuclei fragmentation and cell cycle arrest in the S phase in a concentration-dependent manner K562 cells. K562 cells were left untreated (0 *μ*M) or treated with increasing concentrations of TPGS (10–80 *μ*M) at 37°C for 24 h. After this time, cells were stained with PI. (a) Representative histograms of the cell cycle, showing a sub-G1 section representing DNA fragmentation. (b) Representative histogram of the cell cycle, showing that TPGS induces an increase in the S phase population assessed according to *Materials and Methods*. (c, d) K562 cells were left untreated (0 *μ*M) or treated with 80 *μ*M at 37°C for 24 h. After this time, cells were double-stained with Hoechst (ex. 354 nm, em. 442 nm)/_DiOC6_(3) (ex. 450–490 nm, em. 515 nm) stain. (c) Representative fluorescent merge image of untreated K562 cells with normal nuclei (inset) and high *ΔΨ*_m_. (d) Representative merge image of treated cells with TPGS (80 *μ*M) showing nuclei fragmentation (inset) and loss of *ΔΨ*_m_.

To study the effect of TPGS on CML cells, we first treated human K562 cells with increasing concentrations of TPGS (10–80 *μ*M) for 24 h and then stained the untreated (control) or treated cells with mitochondrial lipophilic dye DiOC_6_(3) and nuclei with PI to assess the viability of the cell in terms of the plasma membrane, *ΔΨ*_m_, and nucleus integrity. Flow cytometry analysis (Figure 1) shows that the viability of K562 cells was concentration-dependently reduced by TPGS (Figure 1(a), quadrant Q3), reflected as a loss of *ΔΨ*_m_ and increased DNA fragmentation (Figure 1(a), Q1+Q4) and typical of apoptosis (Figures 1(b) and 1(c)). Further flow cytometry analysis (Figure 2) shows that TPGS treatments dose-dependently increased fragmentation of DNA (sub-g1 cell population) (Figure 2(a)) and arrested the cell cycle in the S phase, starting at 20 *μ*M TPGS (Figure 2(b)) in K562 cells. Fluorescence microscopy revealed normal nucleus morphology in untreated cells (Figure 2(c)), whereas TPGS (e.g., 80 *μ*M)-treated cells showed typical nuclear fragmentation (Figure 2(d), inset: dot-like highly fragmented nuclei).

### 3.2. TPGS Generates H_2_O_2_, Oxidized the Stress Sensor Protein DJ-1, and Activates Caspase-3 in CML K562 Cells

**Figure 3:**
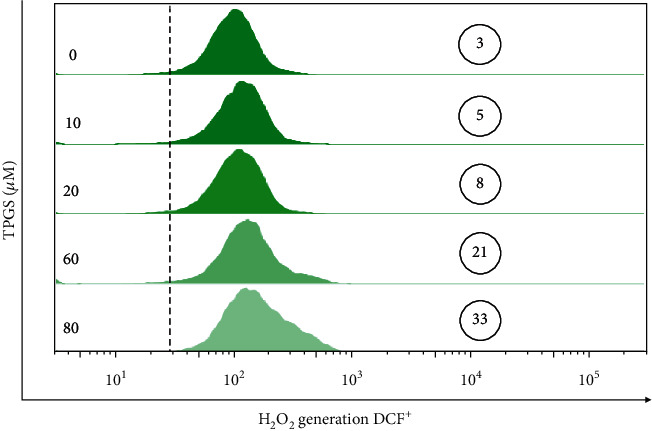
TPGS concentration-dependently induces reactive oxygen species in K562 cells. K562 cells were incubated with increasing concentrations of TPGS (10–80 *μ*M) at 37°C for 24 h. After this time, cells were stained with DCFH2-DA. Representative histograms of the percentage of DCF^+^ cells were assessed according to *Materials and Methods*. The image represents one of three independent experiments. Circled numbers represent the mean percentage ± <5%SD of three independent experiments.

**Figure 4:**
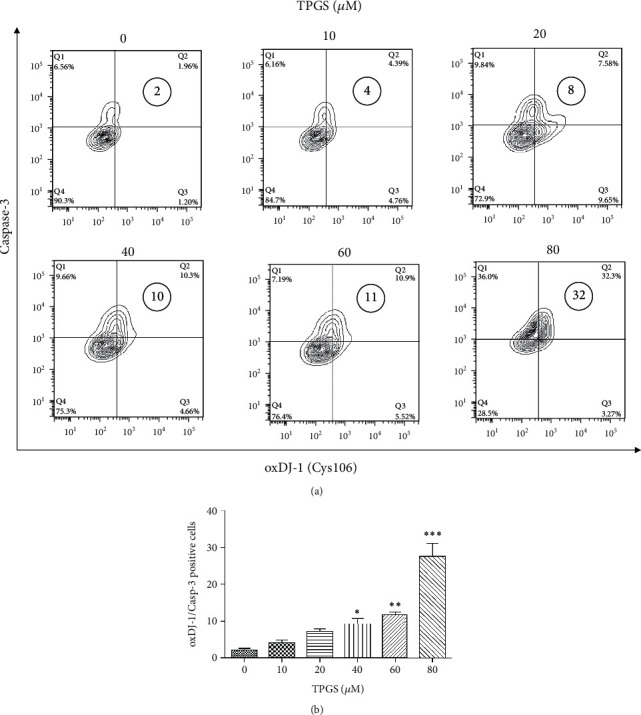
TPGS induces oxidation of DJ-1 (at Cys106 residue) and activation of protein caspase-3 in K562. K562 cells were incubated with increasing concentrations of TPGS (10–80 *μ*M) at 37°C for 24 h. (a) Representative contour plot figures showing the oxDJ-1^+^/caspase-3^+^ double-positive population (Q2). (b) Quantification of percentages of double-positive population cells shown in (a).

Next, we assessed whether TPGS produces ROS in K562 cells.  Figure 3 shows that the fluorescence intensity of H_2_O_2_-sensing fluorescent probe DCFH-DA increased dose-dependently by TPGS in K562 cells. To confirm the generation of ROS (specifically H_2_O_2_) and simultaneous activation of the protein caspase-3 responsible for DNA fragmentation, we used activated caspase-3 and the oxidation of sensor-specific H_2_O_2_-reacting protein DJ-1 (e.g., DJ-1-Cys106-SO_3_) as a probe [31] in cells treated with TPGS (10–80 *μ*M) at 37°C for 24 h. As shown in Figure 4(a), TPGS significantly increased the expression level of protein oxDJ-1 and caspase-3 (Figure 4(b)), albeit (80 *μ*M) had the strongest activation/oxidation effect on K562 cells (e.g., ~32% double CASP3^high^/oxDJ-1^high^ cells vs. ~2% untreated cells).

### 3.3. NAC Reduces TPGS-Induced Apoptosis in CML K562 Cells

**Figure 5:**
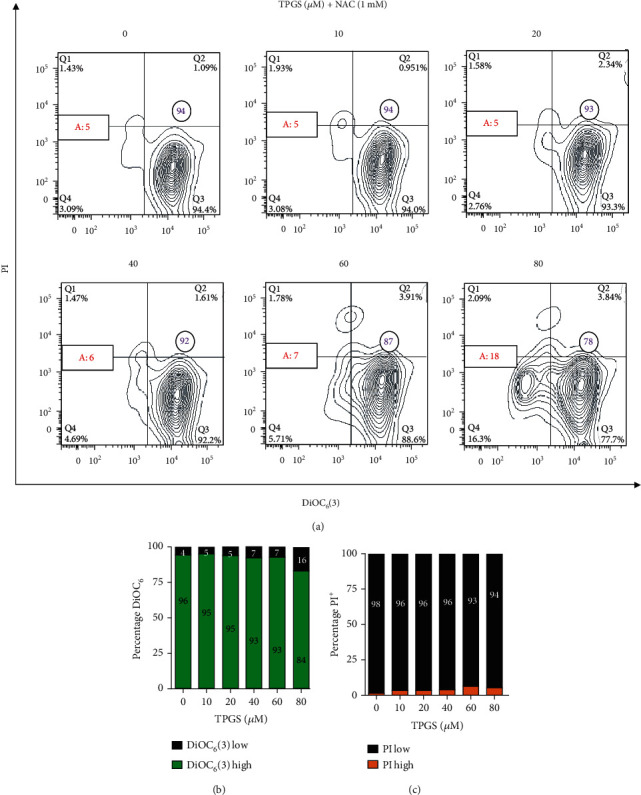
Pharmacological treatment with the antioxidant N-acetyl-L-cysteine prevents the loss of *ΔΨ*_m_ induced by TPGS in K562 cells. K562 cells were left untreated (0 *μ*M) or treated with increasing concentrations of TPGS (10–80 *μ*M) in the presence or absence of NAC (1 mM) at 37°C for 24 h. After this time, cells were double-stained with PI and DiOC_6_(3) stains. (a) Representative contour plot figures showing alive cells (Q3, PI^low^/DiOC_6_(3)^high^), early apoptotic (EA) cells (Q4), late apoptotic (LA) cells (Q1), and unspecified cell death, UCD (Q2). A: represents the percentage of apoptotic cells (Q1+Q4). The purple number in circles represents viable cells, and the red number in rectangles represents total apoptotic cells. (b) Representative histogram of DiOC_6_(3)^low/high^ assessed by flow cytometry. (c) Representative histogram of PI^low/high^ assessed by flow cytometry according to *Materials and Methods*.

To verify that the cytotoxic effect of TPGS was related to OS, K562 cells were also cotreated with the antioxidant compound *N-acetyl-L-cysteine* (NAC, 1 mM) without or with TPGS (10–80 *μ*M). As shown in Figure 5(a), NAC protected K562 cells against TPGS-induced apoptosis compared to untreated cells (Figures 5(b) and 5(c)).

### 3.4. TPGS Induces Upregulation of c-JUN and PUMA but BCL-2/BAX Expression Ratio Was Unaffected in K562 Cells

**Figure 6:**
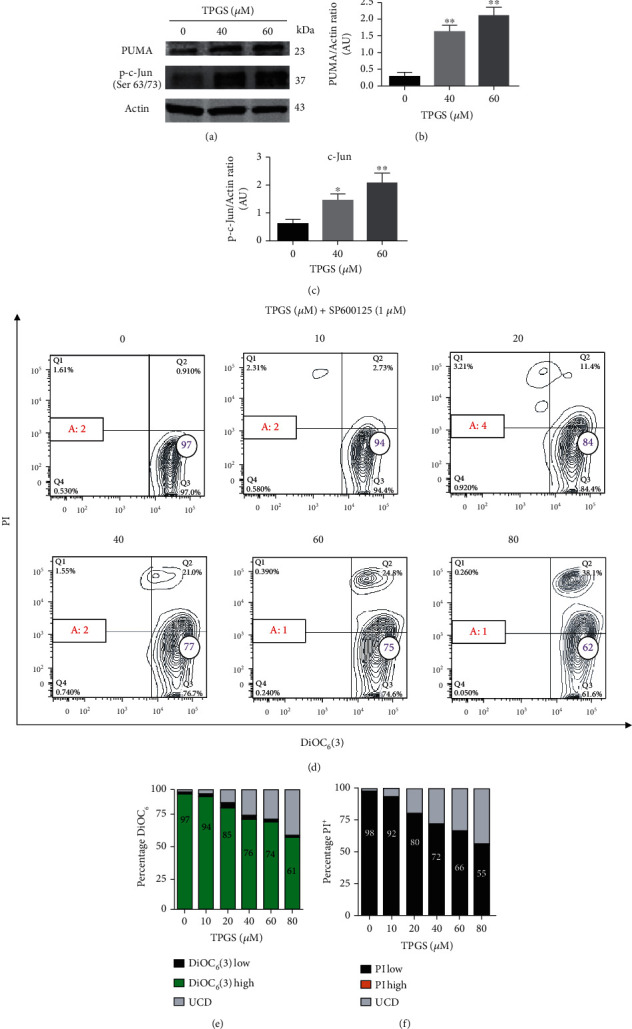
TPGS upregulates the PUMA and phosphorylated c-JUN expression in K562 cells. K562 cells were incubated with TPGS (40–60 *μ*M) at 37°C for 24 h. (a) Protein extracts were blotted with primary antibodies as listed. (b, c) Relative infrared fluorescence of antibodies was expressed as the fold increase relative to that in untreated cells and normalized to actin. Data are expressed as the mean ± SD; ^∗^*p* < 0.05, ^∗∗^*p* < 0.005, and ^∗∗∗^*p* < 0.001. K562 cells were incubated with increasing concentrations of TPGS (10–80 *μ*M) and SP600125 (1 *μ*M) at 37°C for 24 h. After this time, cells were double-stained with PI and DiOC_6_(3) stains. (d) Representative contour plot figures showing alive cells (Q3), early apoptotic (EA) cells (Q), late apoptotic (LA) cells (Q1), and unspecified cell death, UCD (Q2). A: represents the percentage of apoptotic cells as Q1+Q4. The purple number in circles represents viable cells, and the red number in rectangles represents total apoptotic cells. (e) Representative histogram of DiOC_6_(3)^low/high^ and UCD assessed by flow cytometry. (f) Representative histogram of PI^low/high^ and UCD assessed by flow cytometry according to *Materials and Methods*.

**Figure 7:**
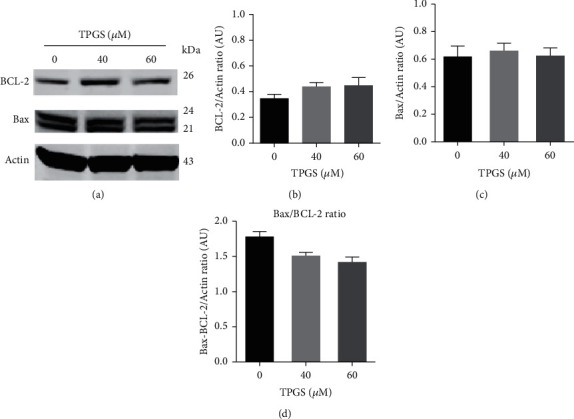
BAX/BCL-2 ratio had no significant changes in the presence of TPGS in K562 cells. K562 cells were incubated with increasing concentrations of TPGS (10–80 *μ*M) at 37°C for 24 h. (a) Protein extracts were blotted with primary antibodies as listed. (b–d) Relative infrared fluorescence of (b) BCL-2, and (c) BAX antibodies were expressed as fold increase relative to that in untreated cells and normalized to actin. (d) BAX/BCL-2 ratio. Data are expressed as the mean ± SD; ^∗^*p* < 0.05, ^∗∗^*p* < 0.005, and ^∗∗∗^*p* < 0.001.

We wanted to further characterize the antitumor molecular mechanism of TPGS in K562 cells. Therefore, we evaluated whether the proapoptotic protein of the BCL-2 family (e.g., BAX, BCL-2, and PUMA), activation of the transcription factor c-JUN, and caspase-3 were involved in TPGS-induced OS cell death. To this aim, K562 cells were exposed to TPGS (e.g., 40 *μ*M and 60 *μ*M) for 24 h. Western blotting analysis revealed that TPGS induced upregulation of protein PUMA (∼2.0-fold increase; Figures 6(a) and 6(b)) and p-c-JUN (∼1.5-f.i.; Figures 6(a) and 6(c)). To confirm the involvement of c-JUN in the TPGS-induced apoptosis process, K562 cells were exposed to TPGS (10–80 *μ*M) with or without JNK inhibitor SP600125 (1 *μ*M). As shown in Figure 6(d), the inhibitor decreased TPGS-induced apoptotic effect (Figure 6(d) (Q1+Q4)) to control values. However, SP600125 provoked an important increase in the percentage of unspecified cell death (UCD) compatible with necrosis cell death (i.e., PI^high^/DiOC_6_(3) high signal, (Figures 6(d) (Q2)–6(f)). Western blotting shows that TPGS induced neither changes in the expression levels of protein BCL-2 (Figures 7(a) and 7(b)) nor BAX (Figures 7(a) and 7(c)) in K562 cells (i.e., BCL-2/BAX ratio, Figure 7(d)) compared to untreated cells (control).

### 3.5. TPGS Increases the Expression Level of and Activates Caspase-3 in K562 Cells

**Figure 8:**
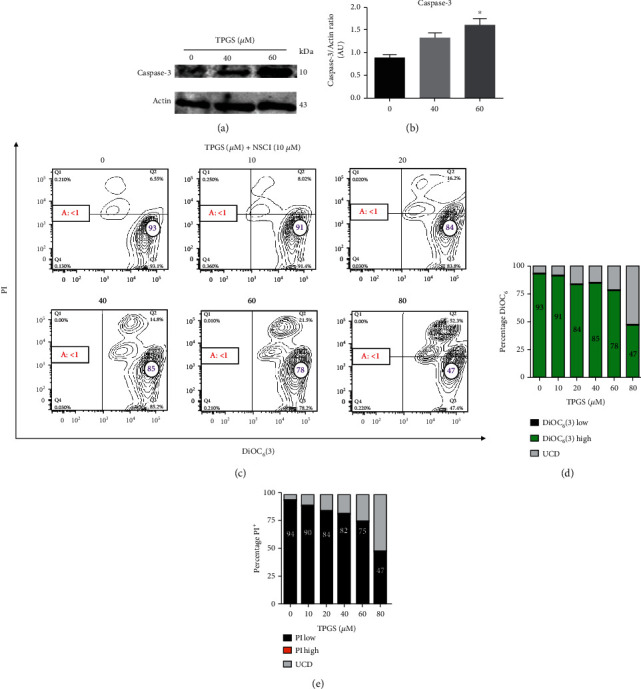
TPGS induces activation of effector protein caspase-3 in K562 cells. K562 cells were incubated with TPGS (40–60 *μ*M) at 37°C for 24 h. (a) Protein extracts were blotted with primary antibodies as listed. (b) Relative infrared fluorescence of antibodies was expressed as the fold increase relative to that in untreated cells and normalized to actin. Data are expressed as mean ± SD; ^∗^*p* < 0.05, ^∗∗^*p* < 0.005, and ^∗∗∗^*p* < 0.001. K562 cells were incubated with increasing concentrations of TPGS (10–80 *μ*M) and NSCI (10 *μ*M) at 37°C for 24 h. After this time, cells were double-stained with PI and DiOC_6_(3) stains. (c) Representative contour plot figures showing alive cells (Q3), early apoptotic (EA) cells (Q4), late apoptotic (LA) cells (Q1), and unspecified cell death, UCD (Q2). A: represents the percentage of apoptotic cells as Q1+Q4. The purple number in circles represents viable cells, and the red number in rectangles represents total apoptotic cells. (d) Representative histogram of DiOC_6_(3)^low/high^ and UCD assessed by flow cytometry. (e) Representative histogram of PI^low/high^ and UCD assessed by flow cytometry according to *Materials and Methods*.

Activation of caspase-3 has been recognized as an essential caspase for DNA fragmentation and morphological changes linked to apoptosis. This feature thus constitutes a marker of this type of cell death process. As shown in Figure 8, TPGS induced a significant increase in the expression level of caspase-3 (1.5-f.i.) according to Western blotting (Figures 8(a) and 8(b)**)**. Accordingly, TPGS significantly activated caspase-3 (e.g., ~11%-68% CASP3^+^ cells) in K562 (Figure 4(a)). To confirm the involvement of caspase-3, cells were exposed to TPGS in the absence or presence of the specific inhibitor NSCI (10 *μ*M). Flow cytometry analysis revealed that NSCI drastically reduced the apoptosis signs in treated cells with TPGS (Figure 8(a) (Q1+Q2)) compared to untreated cells. However, similar to SP600125, the NCSI induced a significant increase in the percentage of UCD (Figures 8(d) (Q2), 6(d), and 6(e)).

## 4. Discussion

**Figure 9:**
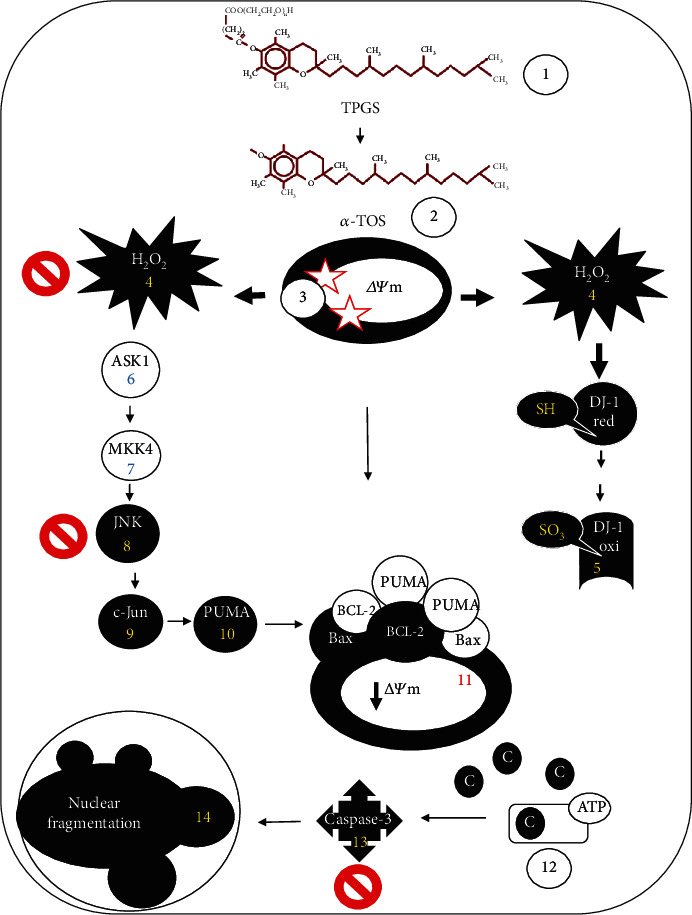
Proposed model of minimal completeness of cell death signaling induced by TPGS as a mechanistic explanation of CML cell demise. The TPGS (step 1) triggers a cell death subroutine in K562, a well-established model of CML. Once TPGS enters the cell, it is metabolically processed by cytoplasmic esterases and converted into alpha-tocopherol acetate (*α*-TOS, s2). This compound targets mitochondrial complex I or II (s3), resulting in an over generation of ROS-H_2_O_2_ (s4). The signaling molecule H_2_O_2_ either oxidized the oxidative sensor protein DJ-1-Cys106-SH into DJ-1-SO_3_ (s5) or indirectly activated prodeath kinases (ASK-1 (s6) and MKK4 (s7)) and JNK (s8), which in turn activate c-JUN (s9). This transcription factor transcribes proapoptotic PUMA (s10), contributing to the permeabilization of the outer mitochondrial membrane (s11). Mitochondrial damage allows the release of apoptogenic proteins such as cytochrome c and ATP, which are responsible for the formation of an apoptosome complex (s12) and activation of caspase-3 protease (s13). This protease in turn activates the endonucleases DFF40/CAD, by cutting the nuclease's inhibitor DFF45/ICAD. Finally, DFF40/CAD causes nuclear chromatin fragmentation (s14), typical of apoptosis. Remarkably, the antioxidant *N-acetylcysteine* (NAC, red stop signs in s4), the specific JNK inhibitor SP600125 (red stop sign in s8), and the specific caspase-3 inhibitor NSCI (red stop sign in s13) block TPGS-induced apoptosis in K562 ratifying the involvement of OS signaling and caspase-3 as end-executor protein in the apoptotic pathway in this leukemia cell line. The TPGS-induced cell death mechanism provides the basis for an oxidative therapy strategy to combat leukemia.

The pharmaceutical industry has used vitamin E TPGS or TPGS (also known as Tocophersolan, PubChem CID: 71406) [32, 33] as a solubilizer, emulsifier, permeation, bioavailability enhancer of hydrophobic drugs, and as an excellent drug deliver agent [34]. Therefore, it has been globally accepted as a safe and nontoxic compound by the principal regulatory agencies [35]. Unsurprisingly, TPGS showed no significant toxic effects in *in vitro* noncancer cells (e.g., peripheral blood lymphocytes) [17] and *in vivo* [19]. However, TPGS has also proved an effective agent to eliminate several cancer cell lines, including ALL cells [17] and *in vivo* tumorigenic cells [19]. Here, we report for the first time that TPGS induces apoptosis in K562 by OS signaling mechanism triggered by H_2_O_2_ and involves the activation of transcription factor c-JUN, upregulation of proapoptotic protein PUMA, followed by loss of *ΔΨ*_m_, activation of caspase-3, and disassembly of nuclei. In contrast to our previous observation showing that TPGS-induced apoptosis in acute lymphoblastic leukemia (ALL) cells by at least 2 complementary death subroutines (i.e., p53-dependent: H_2_O_2_>NF-*κ*B>p53>PUMA and p53-independent: H_2_O_2_>JNK>p-c-JUN>PUMA) that converged in mitochondrial damage, caspase activation, and nucleus fragmentation [17], TPGS induced cell death in K562 by a minimal mechanism involving an H_2_O_2_-dependent and p53-independent pathway, i.e., H_2_O_2_>JNK>p-c-JUN (Ser^63^/Ser^73^)>PUMA>*ΔΨ*_m_ (down)>caspase-3 (up)>dot-like/sphere-like shape DNA fragmentation (Figure 9). Indeed, TPGS generated ROS, specifically H_2_O_2_. Although the cytotoxic mechanism is not yet fully established, one possible explanation stems from its biochemical metabolism. TPGS is a water-soluble amphipathic formulation of D-*α*-tocopherol succinate coupled, through a succinate linker, to polyethylene glycol (PEG) 1000; it is easily taken up into cancer cells. After hydrolysis (e.g., by cytosolic esterases), the fat-soluble D-*α*-tocopherol is then released either couple to acetate, forming the *α*-tocopheryl succinate (*α*-TOS) or *α*-tocopherol (Vit E). While Vit E is harmless to cells, mounting evidence has shown that *α*-TOS induced dissipation of *ΔΨ*_m_, inhibition of mitochondrial complex I or II, and accumulation of ROS [36, 37]. Whatever the mechanism, we found that TPGS significantly increased the DCF^+^ cells reflecting the intracellular generation of ROS (H_2_O_2_). It is well established that H_2_O_2_ acts as a second messenger [38] involved in redox signaling (e.g., activation of apoptosis signal-regulating kinase 1, ASK-1) [39]. Moreover, it has been demonstrated that H_2_O_2_ specifically oxidized the Cys106-SH residue of the DJ-1 protein into DJ-1-Cys106-SO_3_ [31, 40]. We found that TPGS-induced a significant elevation in the oxidized protein DJ-1. Taken together, these findings suggest that H_2_O_2_ is involved in TPGS-induced apoptosis in K562. Furthermore, the antioxidant NAC completely protected K562 cells against TPGS toxic stimuli (Figure 5). Taken together, these observations imply that TPGS produces H_2_O_2_ at mitochondria, and this effect is associated with the dramatic loss of the mitochondrial membrane potential. We also determine that TPGS arrested the cell cycle in the S phase (Figure 2). How does TPGS block the cell cycle in the S phase where the p53—a tumor suppressor gene that normally stops the progression of the cell cycle, is not expressed in K562? We speculate that TPGS can disable important proteins (e.g., cyclin B) involved in the transition from S to the G2-M phase [41]. However, further investigation is needed to clarify this issue.

Previous studies have implicated JNK kinase, phosphorylated c-JUN, PUMA, and caspase-3 in TPGS-induced apoptosis in ALL cells [17]. Here, we confirmed that TPGS induces p-c-JUN and overexpresses PUMA in the K562 cell line. Furthermore, we found that the specific inhibitor JNK SP600125 completely decreased the toxic effect of TPGS according to mitochondrial *ΔΨ*_m_ assay. Taken together, these results suggest that JNK kinase and c-JUN are critical molecules in the cell death process of this cell line. Indeed, once p-c-JUN is activated, this transcription factor transcribes the proapoptotic protein PUMA [42]. In agreement with this view, we found a significant increase in the expression levels of the protein PUMA according to Western blotting (Figure 6). Since p53 is a natural transcription factor that overexpresses PUMA [43, 44] and BAX [45], our observation implies that TPGS can induce apoptosis independently of p53 in K562 [46]. Consequently, we found no changes in the proapoptotic/antiapoptotic protein BAX/BCL-2 ratio [45]. This implies that PUMA is capable of lessening the *ΔΨ*_m_ [47]. Likewise, Western blotting and flow cytometry analyses revealed that TPGS significantly activated caspase-3. Remarkably, the inhibitor NCSI drastically reduced caspase-3 activation in the presence of TPGS. Taken together, these results suggest that JNK/c-JUN, PUMA, caspase-3, and mitochondria are important players in TPGS-induced apoptosis in K562 cells.

## 5. Conclusion

Overall, TPGS is a promising vitamin E synthetic-derived antileukemic agent due to its prooxidant activity and specific targeting of mitochondria. Although its mechanism of action is not yet fully established, our data suggest that TPGS induced apoptosis in K562 by an H_2_O_2_-dependent but p53-independent signaling mechanism. In addition to plasma membrane damage, the TPGS-induced apoptosis mechanism comprises a minimal step initially triggered by H_2_O_2_ and ends up with the activation of protease caspase-3 and nucleus fragmentation (Figure 9). Since TPGS is safe in mice (e.g., a dose of 100 mg/kg through tail-vein injections) [19], TPGS is especially promising for preclinical leukemia research. Considering the present results, detailed biological studies are justified to determine conditions under which the prooxidant properties of TPGS serve to actively burst OS in CML cells.

## Data Availability

The data used to support the findings of this study are available from the corresponding author upon request.
